# Statement of Retraction: Upregulation of serum and glucocorticoid-regulated kinase 1 (SGK1) ameliorates doxorubicin-induced cardiotoxic injury, apoptosis, inflammation and oxidative stress by suppressing glucose regulated protein 78 (GRP78)-mediated endoplasmic reticulum stress

**DOI:** 10.1080/21655979.2026.2745504

**Published:** 2026-11-05

We, the Editors and Publisher of the journal *Bioengineered*, have retracted the following article from publication.

Wang, F., & Han, L. (2022). Upregulation of serum and glucocorticoid-regulated kinase 1 (SGK1) ameliorates doxorubicin-induced cardiotoxic injury, apoptosis, inflammation and oxidative stress by suppressing glucose regulated protein 78 (GRP78)-mediated endoplasmic reticulum stress. *Bioengineered, 13*(1), 844–855. https://doi.org/10.1080/21655979.2021.2013109

Since publication, significant concerns have been raised by a third party about the integrity of the data and reported results in the article.

When approached for an explanation, the authors have not responded to our queries and so these serious concerns remain unaddressed.  

As verifying the validity of published work is core to the integrity of the scholarly record, we are therefore retracting the article. The authors have been informed of this decision.

We have been informed in our decision-making by our policy on publishing ethics and integrity and COPE guidelines.  

